# Comparing the Pressure on the Carpal Tunnel When Using an Ergonomic Pointer Driver and an Optical Alternative

**DOI:** 10.3390/jfmk9040260

**Published:** 2024-12-07

**Authors:** Francisco A. Cholico, José A. Paz, Zaira López, Alfonso Hernández Sámano, Eri Mena-Barboza, José Bernal-Alvarado, Celso Velasquez, Rodolfo Hernández-Gutiérrez, Luis H. Quintero, Mario E. Cano

**Affiliations:** 1 Centro Universitario de los Valles, Universidad de Guadalajara, Carretera Guadalajara-Ameca Km. 45.5, Ameca 46600, Jalisco, Mexico; apolinar.cholico@alumnos.udg.mx (F.A.C.);; 2 Centro Universitario de la Ciénega, Universidad de Guadalajara, Avenida Universidad 1115, Ocotlan 47810, Jalisco, Mexico; 3 Departamento de Ingeniería Física, División de Ciencias e Ingenierías Campus Leon, Universidad de Guanajuato, Loma del Bosque 103, Lomas del Campestre, Leon 37150, Guanajuato, Mexico; 4 Centro de Investigación y Asistencia en Tecnología y Diseño del Estado de Jalisco A.C., Av. Normalistas 800, Guadalajara 44270, Jalisco, Mexico; 5 Centro Universitario de Ciencias Económico Administrativas, Universidad de Guadalajara, Periférico Norte 799, Col. Los Belenes, Zapopan 45100, Jalisco, Mexico

**Keywords:** carpal tunnel, infrared, gestures

## Abstract

**Objectives**: The objective of this paper is to introduce a method to measure the force or pressure over the carpal tunnel indirectly, using a new device to drive the pointer of a computer system. The measurements were compared with those obtained using an ergonomic mouse. Simultaneously, measurements of muscular stress on the digitorum extensor muscle were performed to correlate the applied force against muscle activity. **Methods**: An experimental setup was constructed using an infrared static receiver plus two wearable moving light emitters, which can be displaced inside a rectangular projected region. The pointer functions are performed through two finger gestures, while the hand is naturally extended. A microcontroller was used to communicate with the computer, which works as a human interface device and possesses firmware to associate the position of each light source with the pointer functions. Meanwhile, force and electromyography sensing circuits were developed to transmit and measure carpal tunnel strength and muscular stress. The system was tested on five healthy volunteers, who were encouraged to solve the same computational tasks using this new device and a trademark ergonomic mouse. **Results**: Our results show great differences (greater than one magnitude) between the efforts of the same volunteers performing the same predefined tasks using both pointer controllers. Only when the new device was used did the Pearson’s correlation coefficients show a higher correlation between the effort measured on the carpal tunnel and the muscular activity. **Conclusions**: The optic pointer driver diminishes the strength on the carpal tunnel, causing slightly increased stress on the digitorum extensor muscle.

## 1. Introduction

Carpal tunnel syndrome (CTS) is a common peripheral neuropathy that arises from compression of the median nerve. Frequently, this condition is associated with occupations, age, or gender of people [1,2]. However, the origin of this condition is not entirely known [3]. Several types of research have stated that the pronounced use of mouse and keyboards represents a risk factor for acquiring this health condition [4,5], although other investigations are contradictory [6]. Furthermore, CTS patients could be submitted to surgery and/or physiotherapy sessions [7], and sometimes, they are unable to use a mouse or keyboard. Consequently, they will be unable to carry out computational activities.

The median nerve is distally ramified from the carpal tunnel (CT) to some hand fingers, controlled through the flexor tendons. In some hand motions for holding objects (as the mouse), we have observed some compression on the CT due to hand contractions. That deformation has been modeled in [8], considering various forms of fastening objects. This is probably the leading cause of the recidivism observed in patients with CTS, even when they started using vertical ergonomic mouse pads [9]. Similar negative results were reported in [10]. Nevertheless, positive results were observed only when the ergonomic mouse (EM) use was complemented by education on ergonomic principles in postural hygiene, such as changes in usual behaviors and the importance of extension exercises and work breaks.

During the COVID-19 pandemic, in healthcare clinics, an increasing incidence of neuropathies related to CTS was diagnosed, promoted by long home office activities [11]. It is essential to highlight that no standard method exists to diagnose CTS [12]. As it depends on the clinical history of each patient, the methodologies can range from simple auscultation pressuring the nerves with an object or measuring ultrasonic images plus nerve conductivity tests by electromyograms [13]. Nevertheless, new alternatives to diagnosing CTS have been recently published, aimed at the early detection of symptoms using machine learning or mobile devices [14,15].

Then, it is possible to state that EMs do not maintain free of compression the median nerve, although they conform to the hand shape. Hence, the main aim of this work is the design and development of a new optic pointer driver (called IR Device), which can guarantee the control of a computer pointer, causing minimum stress and compression of the hand, i.e., the median nerve. Because this device does not involve vigorous hand contraction movements, we hypothesize that it causes less stress on the CT. The operation of the IR Device was tested using a biometric detection setup (BDS) on five healthy male volunteers. Moreover, the force applied to the CT area and the muscular stress induced when using the IR Device and solving a predefined task were measured. Both types of measurements were obtained from each volunteer, who performed a predefined computational activity using this IR Device and a commercial ergonomic mouse ELE-GATE G-509. Additionally, elemental comparisons of the measurements were carried out. Our institutional review board classified all the experiments as low risk, and we followed all their recommendations.

## 2. Materials and Methods

### 2.1. Hardware Details of the IR Device and BDS

**Figure 1:**
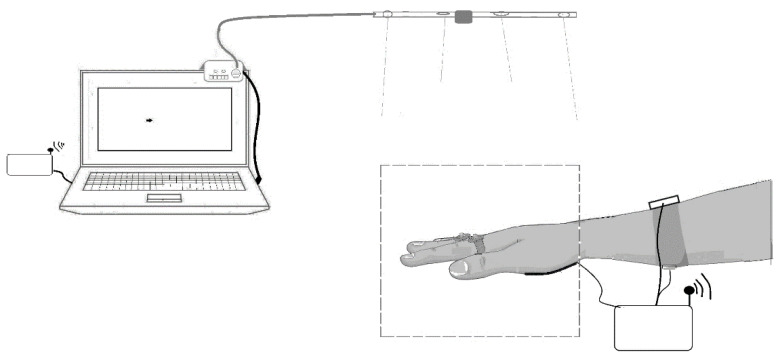
The IR Device and BDS in operation.

The IR Device in operation and the BDS comprise two hardware-design and -development stages. The first one is focused on the emission and detection of infrared sources from the user’s hands, which is controlled using Arduino technology (Arduino LLC, Italy) through its corresponding firmware. The last stage was designed to determine the hand effort and muscular stress-measurement systems. Also, a digital signal controller (DSC) was programmed for data acquisition and signal processing. Figure 1 schematizes the IR Device, which is connected to a USB port of the computer; also, the BDS setup appears connected to the hand and arm of a volunteer. The signals registered by the BDS are transmitted to the computer via RF using a pair of Zig-Bee transmitters (Digi International Inc., Hopkins, MN, USA). All the components of the IR Device and BDS are described in detail below.

**Figure 2:**
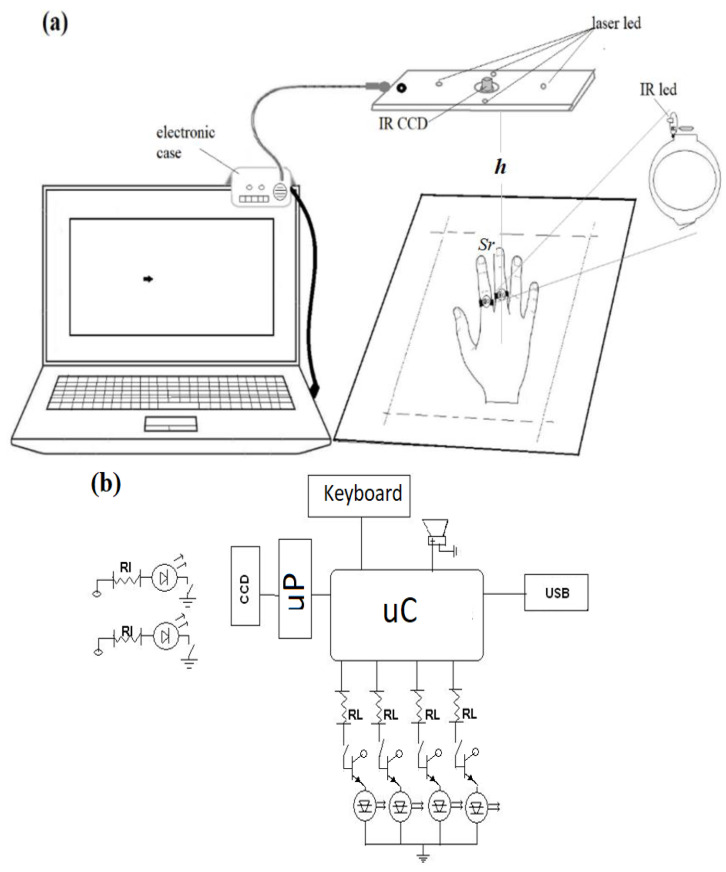
(**a**) The IR Device and (**b**) its schematic diagram.

Figure 2a depicts the IR Device plugged into a laptop via USB. This is mainly composed of an IR emission/reception system. As can be observed, two wearable springy rings with embedded IR emitter LEDs were built and placed in two continuous fingers. Then, while the hand (suitable for ambidextrous) is displaced within a dashed rectangular region, both signals are tracked from the IR receiver camera placed in the upper parallel housing. This IR CCD is connected with the control card placed within a small case, which is fastened, powered, and plugged into the computer to control the pointer.

**Figure 3:**
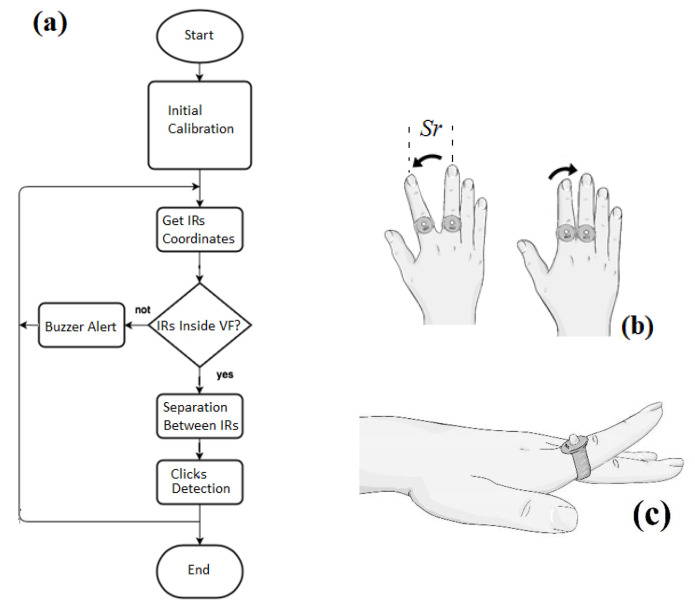
(**a**) The flowchart for tracking the IR sources and shifting the pointer. The gestures associated with (**b**) the right-click and (**c**) the left-click.

Regarding the close-up of a ring in Figure 2a, as is depicted in Figure 2b, each infrared source includes an IR LED of 940 nm of wavelength and 5 mm of diameter. The IR LEDs are connected through a resistance RI with a weak micro switch in the button, which turns the emission on. Both emitters are powered with small coin-like batteries. The projected dashed rectangle is generated with four Laser-LED emitters (5 mm in diameter) coupled with diffraction grids at the tips projecting dashed line patterns. The chosen IR camera is SEN0158 (Zhiwei Robotics Corp, Shangai China), which has 128 × 96 pixels of resolution capability, with 33° and 23° of horizontal and vertical detecting angles, respectively. This camera can detect up to four emitting sources and is connected via I^2^C protocol to the Arduino DUE board. It includes a microcontroller (μC) with a 32-bit ARM CortexM3 microprocessor (μP) (Arm Holdings, Cambridge, England) and uses the pins SCL and SDA for data transfer between the camera and board. Through appropriate firmware, the μC detects the coordinates of each IR source in real time. Each position is transmitted to the PC via USB protocol, and the BDS works as a human interface device HID. The four Laser-LEDs are also enabled using four digital output DIOs through 2N2222 transistors. Another DIO is also used to activate a piezoelectric buzzer transducer as a sonic indicator when any IR emitter has left the dashed rectangle. All the electronic components of the IR emission/reception system are shown and schematized in Figure 2b. Furthermore, the flowchart for tracking the IR sources is depicted in the block diagram of Figure 3a. The processes summarized in the flowchart begin with the calibration of click functions; next, inside an infinite loop, are tracked the IR sources (to shift the pointer), the active status of both IR sources (to enable left-click), and the separation between them (to enable the right-click).

In this work, the conventional right-click function of the pointer is defined with the gesture illustrated in Figure 3b. It consists of a separation motion followed by a union motion of the index finger from the medium one, always maintaining them inside the dashed rectangle. Then, both IR emitters reach the separation *Sr* briefly and immediately return to their natural position. Thus, the firmware of the μC (Figure 2b) interprets this gesture as a right-click instruction. Similarly, the right-click-held option is applied by maintaining the *Sr* between both fingers while displaced on the plane surface and keeping the push switches pressed. Likewise, the left-click functions are activated when the IR source of the index finger is muted. Then, the push interrupter is released when the finger is lifted on the dashed rectangle surface, as illustrated in Figure 3c. Thus, the μC interprets one release and two consecutive releases as one left-click and double left-click, respectively. The gestures involved in the newly defined click functions do not imply the contraction of the hand. Indeed, similar extension motions are recommended for physiotherapy in CTS [10,16,17,18,19] or rehabilitation using video games [20,21].

**Figure 4:**
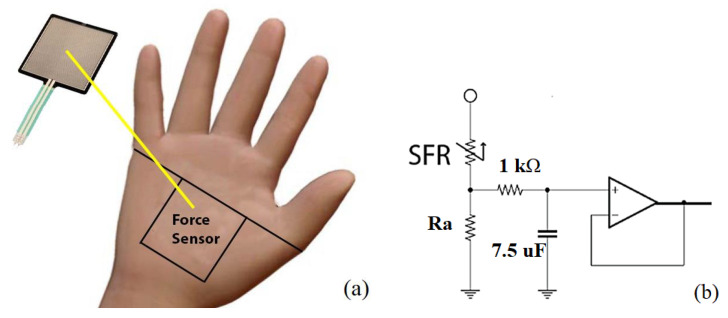
(**a**) Positioning the FSR on the palm and (**b**) its schematic diagram for conditioning the signals.

The flexible force sensitive resistor (FSR) FSR-406 (Interlink Electronics, Inc., Camarillo, CA, USA) is used to indirectly measure the pressure on the CT, which detects the pressure on 16.9 cm^2^ of a rectangle surface. This sensor is a thin, deformable adhesive film. It is placed on the surface of the palm, just on the carpal transversal ligament (located above the median nerve), as is shown in Figure 4a. As is observed, one of its sides is oriented following a diagonal imaginary line connecting the little finger and the thumb. The FSR is configured as a resistive potentiometer; then, it is connected in series with a referenced resistance Ra [22], as is shown in Figure 4b. An RC low pass filter (21 Hz of cut-off frequency) is connected at the FSR output terminal, and it feeds a buffer amplifier (TL 071) to diminish the output impedance.

**Figure 5:**
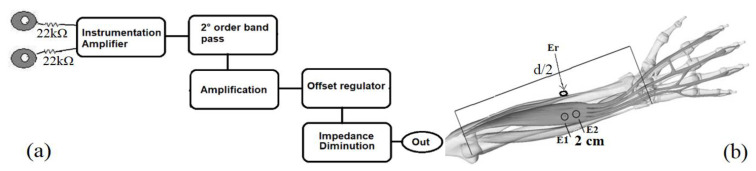
(**a**) The block diagram of the EMG circuit, and (**b**) the placement procedure of electrodes.

As described below, an electromyography EMG system was performed to detect finger movements. Whereas the contraction of the fingers is conducted through its flexor tendons, the digitorum extensor muscle (DEM) produces the extension gesture. When the stress of the DEM vanishes, the hand is contracted, and a possible effort over the CT (i.e., the median nerve) can be detected with the FSR. Hence, surface electrodes are attached to the DEM, and the EMG amplification circuit involves the electronic stages displayed in the block diagram of Figure 5a. Both electrodes are placed on the forearm with a 2 cm separation, as is shown in Figure 5b, and a third electrode is used as a reference sensor connected under the forearm, below the upper electrodes. These three sensors are disposable 3M electrolytic electrodes with 401 mm^2^ of conductive area. The sensed signal through the upper electrodes is amplified *G*_1_ = 101.4 times, using an instrumentation amplifier AD620 configured with a gain resistance *R*_*g*1_ = 492 Ω and applying the gain Equation (1). This increased signal is amplified *G*_2_ = 11 times with a non-inverting amplification, using an operational amplifier circuit (TL071) plus the resistances *R*_1_ = 10 kΩ, *R*_*g*2_ = 1 kΩ and applying the gain of Equation (2). Subsequently, this amplified signal is filtered using a second-order active band-pass filter using two operational amplifiers, and the cutoff frequencies are computed with Equation (3). We chose the bandwidth 20 Hz to 365 Hz. The minimum cutoff frequency is tuned with the components *RHP* = 82 kΩ, CHP = 0.1 μF, and the elements *RLP* = 3.9 kΩ, *CLP* = 0.1 μF are used for the high cutoff frequency. The offset voltage of the filtered signals is regulated using a potentiometer to add a DC voltage from the power supply. The offset control helps to set the baseline of the EMG signals. The resultant signal is then fed to a buffer-like amplifier to diminish the output impedance. A similar circuit was developed to register ECG signals using another bandwidth [23].

(1) $$G_{1}=\frac{49.4\;k\Omega }{R_{g1}}+1$$

(2) $$G_{2}=1+\frac{R_{1}}{R_{g2}}$$

(3) $$f=\frac{1}{2\pi R_{BP}C_{BP}}$$

On the other hand, the conditioned signals provided by the FSR and the EMG circuits are acquired through the analog inputs of the DSC dsPIC33FJ128MC802 microchip (Microchip Technology Inc., Chandler, AZ, USA). In its embedded firmware, all the signals are converted to 12 bits, and only the FSR registers are continuously processed with a Kalman white noise filter using a similar algorithm developed in [24]. The digitized signals are transmitted via RF using Xbee technology (Digi International Inc., Hopkins, MN, USA) to a USB port, where the signals are acquired using RS232 communication in a LabView environment for offline analysis. Thus, while the volunteers solve any computing task using some pointer driver, their biometric signals are stored in real time.

The operation of the IR Device was tested on a group of five volunteers. Then, while the force on the CT was registered, the stress of the finger tendons was indirectly analyzed (sensing the digitorum extensor muscle). The group of volunteers has the following description. They were chosen in an age range of 20 to 35 years, of male gender with right laterality; only healthy volunteers were considered, and their weight and height were not taken into account. In the selection process, we preferred volunteers who daily perform extensive computational activities (more than 7 h a day). From the group of volunteers, three students from the computer-engineering program were chosen, plus two administrative assistants. This sample of participants is characterized because they are subjected to a lot of programming and data-capture work, so the mouse is their main work tool. After a short interview, other volunteers who reported having discomfort in their right hand or a history of upper extremity disease were excluded.

In summary, our dynamic method of measuring the force applied to the palm is related to the CT pressure and the tension of the DEM [25]. Furthermore, according to nervous electroconductivity studies, the pressure variations on the CT are highly correlated with CTS [26,27]. Thus, our methodology makes it possible to determine both variables when a computer activity occurs indirectly: the strength applied on the CT and the stress of the DEM.

### 2.2. Characterization of the IR Device and BDS

**Figure 6:**
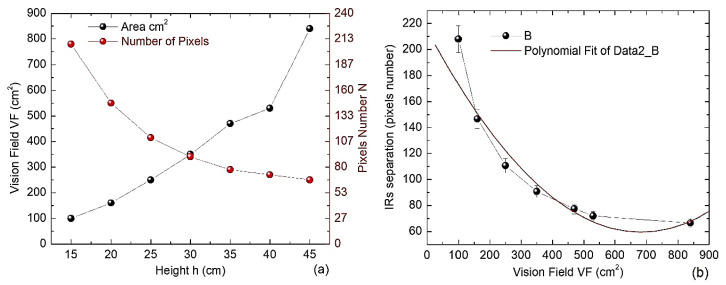
(**a**) The *VF* of the IR chamber (black dots) and separation *S* (red dots) along *h* and (**b**) the correlation between the *VF* and *Sr*.

Due to the spatial resolution of the IR camera and its clearance height *h* from the IR emitter sources, an experiment was conducted to determine the dependence of its vision field *VF* against *h*. For this purpose, both sources were placed on a horizontal plane with millimeter graduation, and 2 cm of separation between them was chosen (the minimum possible). Then, while the position of each source was displayed on the PC screen (with 1920 × 1080 pixel resolution), they were vertically and horizontally displaced to determine the total covered area for each established height *h*. At the same time, the apparent separation “*s*” between the sources observed on the screen was also measured (in pixels). Figure 6a shows the measurements obtained along the interval 15 cm < *h* < 45 cm. As can be observed, the VF (black dots) increases almost linearly before *h* = 40 cm. In contrast, the IR sources appear more separated following a quadratic dependence (red dots, red tick, and labels). Figure 6b shows the dependence between the *VF* and *S*. The fitted line is a quadratic regression. Hence, the *VF* increases linearly when *h* < 40 cm, but both sources appear almost overlapped for the IR chamber, according to a quadratic relationship. Thus, the IR Device is suitable for adjusting *h* and *S* according to the user requirements, but these parameters must be compatible with the selected *VF*, where the IR sources will be displaced.

**Figure 7:**
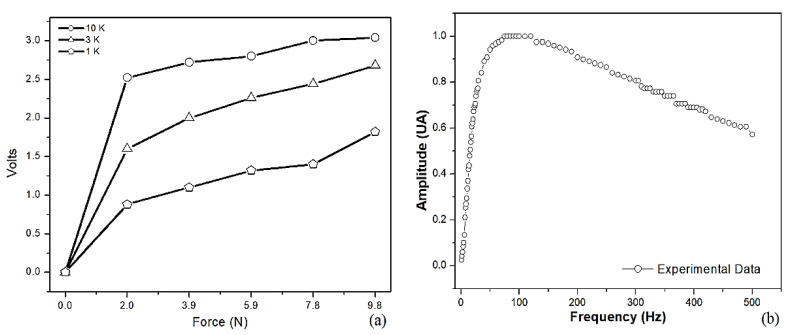
(**a**) Dependence of the applied force on the SFR with its output voltage VOUT; and (**b**) the dependence of the amplitude with the frequency of the EMG circuit.

Another experiment was performed in order to determine the dependence of the FSR output voltage VOUT when a deforming force is applied. Three resistances Rr (see Figure 4b) are provided, taking 1 kΩ, 3 kΩ, and 10 kΩ. The circuit was powered with *V+* = 3.3 V DC using batteries. Additionally, some calibrated reference masses m (0.2, 0.4, 0.6, 0.8, and 1 kg) were placed at the center of the FSR. At the same time, VOUT was registered, and the corresponding forces were estimated using Newton’s law *F* = (9.8 × *m*). This dependence is shown in Figure 7a, and an almost linear behavior above 2N is observed. In our BDS, *Rr* = 3 kΩ was chosen to avoid a fast saturation of the signals, reaching the rate of 0.13 V/N following a linear trend approximately.

On the other hand, the frequency response of the EMG bandpass filter is tested by providing a pure sinusoidal signal at the input. Then, 1 V amplitude is established, and the swept frequency covers the interval 20 Hz < *f* < 500 Hz. This test used a Rigol DG1022 function generator (RIGOL Technologies, Beijing China) and a Rigol DS1074Z oscilloscope (RIGOL Technologies, Beijing China). The latter is employed to obtain both the input and filtered signals. In Figure 7b, the dependence of the measured amplitude along the frequency is displayed. This plot shows a fast amplitude increase, with a minor cutoff frequency of 23 Hz, followed by a small constant amplitude region in 72 Hz < *f* < 122 Hz. In the end, there was a slight decrease with a higher cutoff frequency of 370 Hz. This bandwidth established is very close to the one used in other experimental works [23].

### 2.3. Configuring the IR Device and the Test Interface

After the electronic work, assembling, and characterization, both pointer drivers are ready to be tested simultaneously with the BDS. To carry out this testing, the next procedure was necessary. The FSR and EMG devices were initially placed on the respective hands and arms of each volunteer, as detailed in Figure 4a and Figure 5b, respectively. Later, the rings with the IR emitters were placed on their index and mean fingers, preferably, as illustrated in Figure 2a. Then, a switch on the small case was activated to project the dashed rectangle, delimiting the corresponding *VF*. Previously, the height was established at *h* = 29 cm, which implies a *VF* = 350 cm^2^, and thus, the minimum separation of both IR sources was 2 cm. Then, an open scissor motion of the fingers reaching *Sr* = 3 cm is recommended to avoid failed right-click applications, which is approximately *S* = 140 pixels of separation observed on the screen. After 5 min of adaptation and training, the users can drive the pointer with good precision on the screen, like using a “big touchpad”.

**Figure 8:**
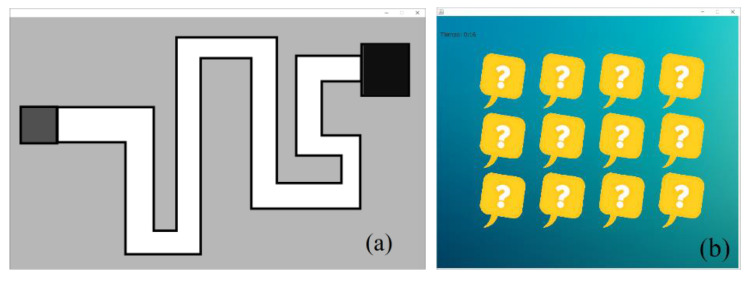
Screen-printing of (**a**) the first and (**b**) second tasks, respectively.

Two computing tasks were programmed using routines in a Java environment to probe the IR Device and BDS. These activities involved using all the pointer resources, which were solved for all the volunteers. The intervention of the volunteers consists of the next activities. As the main subject of the first task is the tracking and motion of the pointer following a predefined pattern. Figure 8a shows a screen print of the interface. The goal of users is to transport the cursor from one dark point until it reaches the other one, while keeping it inside the white area. Likewise, the subject of the second task is to provoke several times the use of one right-click and double left-click. Then, in Figure 8b, a memory card matching game is displayed, which must be solved for all volunteers. One right-click was used to open a selected letter, and a double left-click was used to select the pairs.

Hence, while the volunteers perform both tasks using each pointer driver, the BDS will measure the next variables. The force applied on the CT in real-time using the setup is displayed in Figure 4a,b; the bioelectric signal produced by the DEM using the electronic circuit is depicted in Figure 5a,b.

### 2.4. Data Processing and Statistical Analysis

All measurements are stored and analyzed offline using the Origin 6.0 software. The data obtained from the force on CT were processed as follows. Initially, the force was measured when the hand was resting on each mouse, and the average value applied over 10 s was estimated. For subsequent measurements, as all the waveforms of force have an irregular shape, we will determine only the threshold values applied in a defined time interval.

The data obtained from the EMG signals were processed as follows. As the waveforms of the EMG signals are characterized by a regular baseline, the baselines were approximated to the mean values [28], and the amplitudes were approximated to the corresponding standard deviations [29].

The correlation between the forces on CT and EMG amplitudes on the DEM of each volunteer are estimated by computing the Person’s correlation coefficient using linear fits to the data.

## 3. Results

### 3.1. Initial Tests

**Figure 9:**
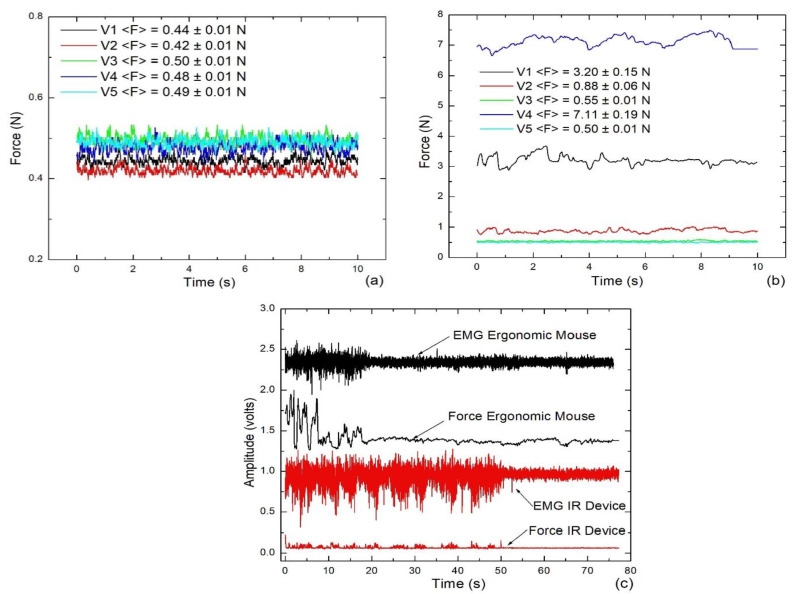
Typical measurements of the force at rest condition using (**a**) the IR Device and (**b**) the EM; (**c**) the measurements of force and EMG using both pointer controllers.

Before the solution of the later computing tasks, measurements of force and EMG of each volunteer were registered only by holding up the IR Device and the EM, defined as rest or hand relaxed. In that condition, the typical signals measured from the CT are depicted in Figure 9a,b. All the experimental data of each plot were averaged by computing the arithmetic mean.

On the other hand, to visually show the possible dependence of the force measurements on the EMG signals, a preliminary experiment was conducted with a volunteer encouraged to perform random clicks and motions of the pointer for a predefined time. This was the first time that volunteers were able to experience using both pointer controllers, and it was also the first computing activity performed before completing the two predefined tasks. Figure 9c depicts the simultaneous amplitudes of FSR and EMG (the offset voltage was deliberately set), where the measurements obtained using the EM are plotted with black lines, and the plots with red lines are the measurements registered using the IR Device.

### 3.2. Solving the Predefined Computing Tasks

**Figure 10:**
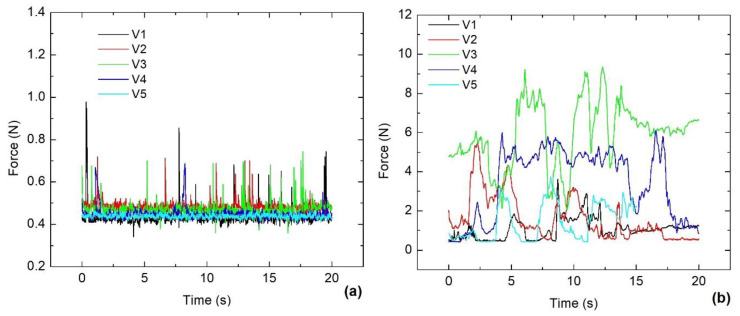
The force measurements during the first task using (**a**) the IR Device and (**b**) the EM.

The volunteers solved the first task, and the corresponding force measurements using the IR Device and EM are displayed in Figure 10a and Figure 10b, respectively.

Concerning the corresponding EMG measurements obtained at the same time interval, Table 1 summarizes all the SDs and the threshold values of force.

**Figure 11:**
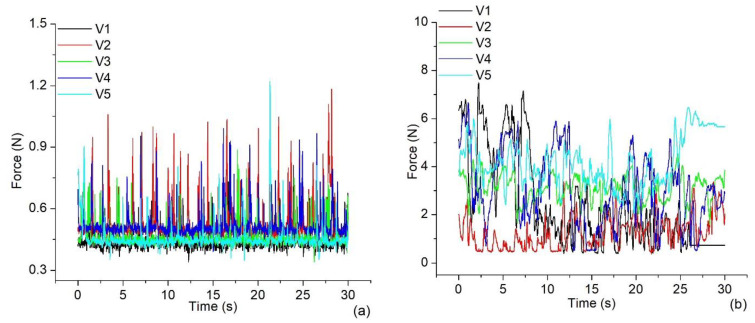
Measurements of the force during the second task completion using (**a**) the IR Device and (**b**) the EM.

The volunteers solved the second task, which involves the left and right clicks combined with the shifting of the pointer; the number of prominent spikes in the force amplitudes of all the volunteers was higher than that observed completing the first task. Figure 11a,b show the signals registered using the IR Device and the EM, respectively.

Regarding the corresponding EMG measurements during the second task, Table 2 summarizes all the estimated SDs associated with using each pointer controller. Additionally, it includes all the corresponding threshold values of force.

**Figure 12:**
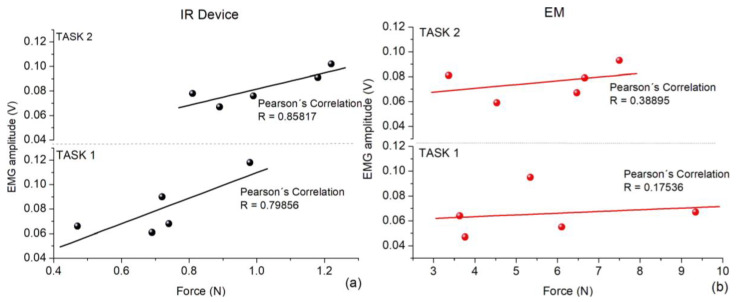
Pearson’s correlation coefficient of the threshold of force with the EMG amplitude of each volunteer solving both tasks using (**a**) the IR Device and (**b**) the EM.

Figure 12a,b depict the Pearson’s correlation coefficients between the forces on CT and EMG amplitudes on the DEM of each volunteer. This parameter was estimated for both tasks using both pointer drivers; the linear traces are regressions fitted to the data. The dark dots were obtained using the IR Device, and the red dots using the EM.

## 4. Discussion

When the hands of the volunteers were resting on each pointer driver, high differences in the stress applied on CT were exhibited, as depicted in Figure 9a,b. The average forces applied on the CT area using the IR Device are within the interval from 0.42 to 0.50 N, but using the EM; this force interval covers from 0.5 to 7.11 N, an order of magnitude larger. Although we observed high strengths on the CT when the hands are resting on the EM, these static efforts cannot be associated with CTS. As was shown in Kubo et al. [25], only variable efforts can cause the CTS. The efforts changed when random movements were performed. The waveforms registered are quite different from those observed at rest condition. For example, when using EM for 20 s, the recorded signals FSR and EMG showed higher amplitude increments simultaneously, forming many peaks of force and voltage, respectively. Later, those amplitudes were significantly diminished when the hand became at rest (for 55 s) and following a baseline, which is very similar to the one observed in Besomi et al. [30]. Additionally, when using the IR Device for 50 s and releasing for 25 s, the same behavior is observed in the amplitudes of FSR and EMG (red line plots). Then, the observations obtained through this initial experiment allowed the necessity of defining a criterion to compare the force and muscular stress applied for a volunteer, using both pointer drivers to solve the same computational task. Analyzing again the measurements of Figure 9c and neglecting the offsets, during the first 20 s, 0.75 N is the threshold of force applied on EM, and 0.07 V is the SD of the EMG. Both magnitudes diminish to 0.15 N and 0.03 V when the hand relaxes on the EM. Concerning the use of the IR Device, during the first 55 s, 0.17 N of threshold and 0.11 V of SD were reached. Those values decreased to 0.01 N and 0.03 V when the hand relaxed on the IR Device. Hence, when the IR Device is displaced, the increment of the EMG is the highest, but the strength is the lowest.

When the volunteers solved the first task, the waveforms registered in Figure 10a,b were quite different from those observed at rest conditions as depicted in Figure 9a,b. Using the IR Device, the force shows an almost well-defined baseline trend in all volunteers. Some prominent peaks are associated with small transients of effort on the CT, but the threshold values were never higher than 1 N. In contrast, even when the same task was performed using EM, the baseline trend disappeared, and a few times, the intensities were less than 1 N. Additionally, in the corresponding EMG signals (Table 1), all the SDs exhibit a considerable increase compared with the measurements obtained with the hand relaxed. Indeed, the highest increments of SDs were observed when the IR Device was used and this is caused because it requires having the hand extended, as was observed by Leijnse et al. [31].

When the volunteers solved the second task, the signals displayed in Figure 11a show a baseline trend using the IR Device again. The higher thresholds of force registered (volunteers V2 and V5) were very close to 1.2 N. In contrast, the forces measured using the EM depicted in Figure 11b show many transients on the CT, applying intensities significantly greater than those applied using the IR Device. The shapes of the plots are entirely different from those observed when the hand was relaxed, as depicted in Figure 7b. In any case, the threshold values exceed the 9.3 N registered when solving the first task. In the corresponding EMG signals, only three volunteers (V2, V3, and V5) using the IR Device show the highest SD values (Table 2) compared to the other two (V1 and V4) who used the EM. It was observed again that most volunteers reached higher increments of SDs when the IR Device was used. This is the same behavior observed in Leijnse et al. [31].

The Pearson’s coefficients depicted in Figure 12a for both tasks using the IR Device show a relatively good correlation, reaching R = 0.80 and 0.86 in round numbers, respectively. Those statistical parameters show good concordance with the correlation observed in studies about EMG and hand-grip strength by Trinidad-Fernandez et al. [32]. Both are values close to the ideal value R = 1.0. In contrast, as shown in Figure 12b, we estimated R = 0.17 and 0.39 for tasks solved using the EM. This statistical result reinforces the statement systematically observed above; the use of the IR Device is related to a lower effort on CT but a higher stress on DEM. In contrast, when using EM, that statement does not have sufficient statistical support.

In concordance with the measurements of the biomechanical signals using both pointer controllers, the great forces applied to the CT using the EM device are clear, even when the hand is relaxed on the mouse. The big differences in the mean force applied for holding the two different mouse drivers can be associated with the surface of the hand. Mainly because the most extensive hands suffer more significant deformation holding the EM, promoting higher effort on the CT. According to the predictions of Bullock et al. [8], this is an expected behavior considering the hand deformations when fastened to different objects. These high forces registered to drive the EM also show good concordance with the results obtained by Schmid et al. [9] and Trillos-Chacón et al. [10], when using different computing peripherals.

In general, the pointer driving on the screen without activating the click functions significantly increases the force compared with the effort made at rest. Comparing the strength applied using both pointer drivers during the first task, the relative threshold minimal value was 3.7 times higher using the EM (this is $\frac{3.63\;N}{0.98\;N}$ reached by V1). From another point of view, significant differences can be highlighted when comparing the strength necessary to drive the pointer with the strength applied to support the weight of an equivalent mass. For example, analyzing the volunteer V3, a threshold of 9.3 N was registered using the EM, and only 0.7 N was registered using the IR Device. According to the calibration presented in Figure 7a, the effort involved in using the IR Device is equivalent to supporting a mass of up to 0.071 kg and 0.92 kg using the EM. Then, the use of EM represents hard work for long-time computing activities. According to Wolf et al. [33], this excessive work could define a trigger for muscular fatigue that puts the mobility of a user at risk. Moreover, according to the results registered during the first task, the shift of the pointer using the EM promotes the most vigorous effort on the CT and a lesser stress increment on the DEM, and vice versa when the IR Device is used. When the IR Device was used, it allowed a higher increase in the EMG signals measured from the DEM. This is possible because the hands remain beyond their natural extension for longer; according to the measurements of Ramana et al. [34], natural extension does not need much muscular effort.

On the other hand, slight additional increments of the thresholds of the force are necessary for combining motions of pointer shifting with click functions using the IR Device. The relative threshold minimal value was only 2.9 times higher using the EM (this is $\frac{3.37\;N}{1.18\;N}$ reached by V2). With the EM, the click activation involves many impulses observed as signals with fast force transients. Using the IR Device, the force followed a baseline with small peaks showing threshold values of force less than 1.2 N. Then, the IR Device only induces short and weak transients of forces on the CT, and a few times, the force reached higher values than when the hand was relaxed. Hence, the gestures depicted in Figure 3b,c increase the strength of the CT in a few decimals of N, which is related to an insignificant hand deformation. As expected, this behavior contrasts with the hand deformation modeled in Bullock et al. [8] to fasten objects, but that deformation was evident using the EM. Most volunteers applied an additional strength increment when combining shifting and clicking on the EM. Under these conditions, the increasing amplitude of the EMG was principally observed using the IR Device.

Although the IR Device offers a considerable decrease in CT pressure, it may be limited for use by people who perform extremely strenuous computing activities; for example, gamers who in a gaming session may perform hundreds of pointer controller operations per minute, including very sudden hand movements [35].

## 5. Conclusions

The use of the IR Device diminishes the effort on the CT, but it is correlated with an increase in stress on the DEM. Our experimental results reinforce the affirmations previously reported in several works about the possible adverse effects on the CT induced by the pointer pad drivers, even when using an ergonomic device. Implicit uncontrolled and repetitive hand contractions principally promote it. Although this work consisted of a pilot study, many more measurements are needed using a larger sample of volunteers with some common characteristics (probably including volunteers diagnosed with CTS.). Its application to other types of tasks that are much more representative of everyday computing tasks should also be considered. Based on our results, we suggest using optical alternatives to carry out exhaustive computer work to avoid the incidence or recidivism of the SCT.

## 6. Patents

The following patent resulted from the work reported in this manuscript MX/u/2019/000298.

## Figures and Tables

**Table 1:** The SD estimations of the EMG signals measured during the first task and the threshold values of the force. In addition, the SDs of the EMG estimated with the hand relaxed.

Volunteer	IR Device SD (V)	Threshold (N)	EM SD (V)	Threshold (N)	IR Device Rest SD (V)	EM Rest SD (V)
V1	0.118	0.98	0.064	3.63	0.019	0.029
V2	0.090	0.72	0.095	5.34	0.031	0.047
V3	0.068	0.74	0.067	9.34	0.031	0.040
V4	0.061	0.69	0.055	6.10	0.035	0.031
V5	0.066	0.47	0.047	3.76	0.039	0.044

**Table 2:** The SD estimations of the EMG signals measured during the second task completion and the corresponding force threshold values.

Volunteer	IR Device SD (V)	Threshold (N)	EM SD (V)	Threshold (N)
V1	0.078	0.81	0.093	7.50
V2	0.091	1.18	0.081	3.37
V3	0.067	0.89	0.059	4.53
V4	0.076	0.99	0.079	6.66
V5	0.102	1.22	0.067	6.47

## Data Availability

The data used to support the findings of this study are available from the corresponding author upon request.
